# Supplementary material for: National Dog Survey: Describing UK Dog and Ownership Demographics
Source: Animals (Basel). 2023 Mar 16;13(6):1072. doi: 10.3390/ani13061072 (PMC10044414; doi:10.3390/ani13061072)
Supplement: Supplementary file 1 [file animals-13-01072-s001.zip › animals-2279877-supplementary.pdf]

Utilising A National Survey To Describe Dog And Ownership Demographics in The UK

Supplementary Materials

Table S1. Data counts for dog demographic data.

Variable	N	Percent
**Dogs age**		
Under 1	42,519	9.7
1-3	90,591	20.6
>3-6	107,990	24.5
>6-9	91,377	20.7
>9-12	68,899	15.6
>12	39,047	8.9
**Dogs sex and neuter status**		
Female spayed	153,182	34.8
Female unneutered	49,328	11.2
Male neutered	164,986	37.5
Male unneutered	72,927	16.6
**Breed status**		
Purebred	255,416	58.0
Known crossbreed	121,548	27.6
Unknown cross/mixed breed	63,459	14.4
**Acquisition Source**		
General selling websites, e.g. Gumtree	36,704	8.6
Pet selling websites, e.g. Pets4Homes	90,761	20.6
Social media	20,276	4.6
Kennel Club's breeder webpages	27,373	6.2
Breed group websites	11,563	2.6
Individual breeders' websites	15,275	3.5
UK-based charity/rehoming centre websites	53,418	12.1
Overseas charity/rehoming centre websites	12,015	2.7
Visit to breeder	25,655	5.8
Visit to UK-based charities/rehoming centres	19,086	4.3
Local press, e.g. newspaper adverts	7,729	1.7
Local adverts, e.g. shop windows	2,370	0.5
Through family or friends	80,124	18.2

Through people in the local community/word of mouth	10,376	2.4
Other	55,011	12.5
Missing data	1051	0.2
**Year of acquisition**		
Pre-2000	242	0.1
2000-2009	30,000	6.8
2010	16,321	3.7
2011	18,424	4.2
2012	20,355	4.6
2013	23,198	5.3
2014	26,108	5.9
2015	29,138	6.6
2016	32,742	7.4
2017	35,392	8.0
2018	43,719	9.9
2019	49,999	11.4
2020	62,151	14.1
2021 (up to Oct 2021)	52,273	11.9
**Age at acquisition**		
Under 1	287,830	65.4
1-3	73,636	16.7
>3-6	31,374	7.1
>6-9	12,188	2.8
>9-12	4,241	1.0
>12	967	0.2
Missing data	30,187	6.9
**Cost of dog**		
No cost	58,112	13.2
< £100	31,772	7.2
£100-250	86,193	19.6
£251-500	91,761	20.8
£501 - 1000	100,562	22.8
£1001-2000	52,677	12.0
£2001-3000	16,423	3.7
£3001-4000	2,302	0.5
£4001 – 5000	329	0.07
> £5000	292	0.07
**Registered with vet?**		
Yes	432394	98.2
No	8,029	1.8
No	*1462*	*18.2*
No but plan to	*3276*	*40.8*
No but will if unwell	*3291*	*41.0*

**Payment of vet fees**		
Veterinary insurance	285,076	64.7
Savings	195,814	44.5
With support from friends or family	32,834	7.5
With support of a charity	8,458	1.9
Don't know	11,944	2.7
Prefer not to say	12,677	2.9

Table S2. Data counts for owner demographic information.

	Dog owners	
	N	%
**Gender**		
Male	65,311	18.4
Female	261,058	73.7
Non-binary	659	0.19
Prefer not to say	4,095	1.2
Missing	22,923	6.5
**Age**		
18-24	16,273	4.6
25-34	43,393	12.3
35-44	48,788	13.8
45-54	88,338	25.0
55-64	82,642	23.3
65-74	40,186	11.4
75+	7,863	2.2
Prefer not to say	3,641	1.0
Missing	22,922	6.5
**Region**		
Crown dependencies	244	0.1
East of England	34,188	9.7
East Midlands	19,829	5.6
Greater London	20,060	5.7
Northeast England	15,697	4.4
Northwest England	37,959	10.7
Northern Ireland	6,070	1.7
Scotland	27,125	7.7
Southeast England	44,785	12.6
Southwest England	34,946	9.9
Wales	16,491	4.7
West Midlands	28,867	8.2

Yorkshire and the Humber	30,178	8.5
Missing data	37,607	10.6
**Overall household size (adults & children)**		
1	78,192	22.1
2	123,475	34.9
3	59,341	16.8
4	48,624	13.7
5	12,257	3.5
6	4,646	1.3
>6	3,331	0.9
Missing data	24,180	6.8
**Children in household**		
Infants		
Yes	29,251	8.3
1-2	*24,761*	*84.7*
3-4	*3,578*	*12.2*
>4	*912*	*3.1*
No	324,795	91.7
Children		
Yes	69,289	19.6
1-2	*62,977*	*90.9*
3-4	*5,400*	*7.8*
>4	*912*	*1.3*
No	284,757	80.4
**Household types**		
Adult only	275,159	74.5
*Single Adult**	*22,922*	
*At least one adult***	*83,183*	
Multiple Adults	*169,054*	
Adult and children	63,384	17.2
Adult and infants	22,786	6.2
Adult, children, and infants	7,858	2.1

*Single adults - those that ticked 0 people in response ** Could either be a single adult or 2 adults - Respondent ticked one adult only however due to survey limitations where it appeared to be unclear to respondents to include themselves initially, this could include both respondents reporting just themselves and answered correctly or incorrectly with 2 adults and neglected to include themselves.

Table S3. – Numbers of females and males aged between 1-2 years that are neutered.

	Female	Male
**Intact**	16647	24165
**Neuter**	11009	13820

Table S4. – Numbers of females and males aged greater than 10 years that are neutered.

	Female	Male
**Intact**	2790	7185
**Neuter**	31501	32763

Table S5. Number and proportions of female and male dogs neutered and intact by year of age.

	Female			Male		
**Age (Years)**	**Intact**	**Neutered**	**Proportion of dogs intact**	**Intact**	**Neutered**	**Proportion of dogs intact**
1	11005	2347	82.4	15763	3271	82.8
2	5642	8662	39.4	8402	10549	44.3
3	2397	8612	21.8	4658	9770	32.3
4	1787	9037	16.5	3567	10323	25.7
5	1397	8766	13.7	2920	10333	22.0
6	1176	8399	12.3	2495	9968	20.0
7	1063	8195	11.5	2277	9399	19.5
8	926	8281	10.1	2101	9196	18.6
9	786	7998	8.95	1864	8639	17.7
10	703	7313	8.77	1724	7903	17.9
11	586	6568	8.19	1577	6904	18.6
12	519	5779	8.24	1328	6092	17.9
13	372	4445	7.72	961	4569	17.4
14	271	3453	7.28	734	3318	18.1
15	168	2084	7.46	449	2151	17.3
16+	171	1859	9.19	412	1826	22.56

Table S6. – Cost paid for the top 25 most common dog breeds across all years.

Breed	No cost	<£100	£100–£250	£251–£500	£501–£1000	£1001–£2000	>£2000
Beagle	11.53	4.48	17.69	20.54	31.65	11.90	2.21
Bichon frise	15.35	3.39	16.12	36.06	22.04	5.27	1.78
Border collie	13.54	10.64	30.80	23.79	14.87	5.93	0.43
Border terrier	8.19	2.34	8.34	34.48	35.65	7.72	3.28
Boxer	8.76	1.73	10.42	12.44	32.10	25.71	8.84
Cavalier king Charles spaniel	9.63	1.33	7.71	23.32	37.28	12.93	7.80
Cocker spaniel	9.19	1.36	6.02	22.46	39.77	13.83	7.37
Cockerpoo -	4.95	0.42	2.31	10.15	44.19	23.92	14.05
Dachshund (miniature smooth haired)	7.57	1.05	3.75	5.49	27.94	39.11	15.10

French bulldog	13.49	0.72	5.12	6.81	19.69	37.25	16.92
German shepherd dog	11.44	3.85	16.29	17.92	29.97	17.23	3.29
Golden retriever	7.81	1.05	4.50	10.51	36.75	28.82	10.56
Greyhound	11.37	15.60	66.43	5.71	0.47	0.34	0.07
Jack Russell terrier	19.84	12.82	39.72	17.60	6.22	3.69	0.11
Labradoodle	6.33	1.20	5.35	16.49	37.45	20.99	12.19
Labrador	10.39	2.55	9.70	23.73	34.75	13.50	5.39
Lhasa apso	15.74	3.57	11.01	39.13	25.31	4.91	0.34
Miniature schnauzer	6.20	0.84	3.42	14.41	50.61	17.34	7.18
Pug	12.59	1.25	6.74	14.14	39.24	24.52	1.53
Shih tzu	15.18	2.96	12.99	38.85	21.98	7.24	0.80
Spaniel (English springer)	13.00	3.47	15.90	34.95	21.53	9.35	1.80
Staffordshire bull terrier	21.56	13.22	30.78	15.37	11.49	5.61	1.97
West highland white terrier	10.41	3.22	12.03	37.67	28.40	5.33	2.94
Whippet	11.52	5.30	16.79	25.79	26.92	11.66	2.03
Yorkshire terrier	19.31	4.87	19.01	35.68	14.24	5.97	0.92

Table S7. – Cost paid for dog broken down by year of acquisition and source.

		**No cost**	**< £100**	**£100–250**	**£251–500**	**£501–1000**	**£1001–2000**	**>£2000**
**Year of acquisition**	**Source**							
2016	Breeder	1.94	0.77	3.11	18.89	58.37	15.19	1.75
	Charity	7.53	19.17	59.69	11.93	1.13	0.33	0.21
	Commercial	2.55	4.22	16.94	36.77	34.39	4.46	0.68
	Personal	40.51	4.56	13.08	21.59	16.95	2.71	0.61
2017	Breeder	2.20	0.77	2.63	14.56	56.57	20.86	2.42
	Charity	6.75	16.22	57.85	17.52	1.23	0.25	0.18
	Commercial	2.41	3.24	14.16	34.18	38.29	7.07	0.66
	Personal	38.99	4.66	11.52	19.92	20.27	4.01	0.62
2018	Breeder	2.05	0.44	2.12	13.20	52.24	26.97	2.98
	Charity	6.91	13.80	56.45	20.97	1.38	0.17	0.32
	Commercial	2.47	2.27	11.77	31.86	41.11	9.69	0.82
	Personal	39.87	3.38	9.46	17.71	22.50	6.28	0.79
2019	Breeder	2.23	0.54	2.05	9.50	46.39	35.26	4.03
	Charity	6.24	9.67	53.39	28.44	1.95	0.24	0.07

	Commercial	2.04	1.96	8.88	27.66	44.11	14.34	1.01
	Personal	39.90	2.91	8.42	16.59	23.17	8.06	0.95
2020	Breeder	1.96	0.27	1.27	3.90	24.84	48.93	18.84
	Charity	5.77	4.84	35.02	48.26	5.43	0.43	0.25
	Commercial	1.51	0.85	3.09	10.39	27.57	37.21	19.38
	Personal	30.68	2.00	6.36	12.57	23.15	19.49	5.74
2021	Breeder	2.73	0.21	1.01	1.89	11.09	48.03	35.05
	Charity	5.27	3.08	23.99	53.79	12.90	0.55	0.42
	Commercial	1.48	0.51	1.75	6.14	22.39	43.04	24.68
	Personal	27.49	1.09	3.34	7.70	17.57	30.81	12.00

NB. Values are the percentage of all dogs acquired each year which fall into each price category. Breeder = breeder website, breed group website, kennel club website breeder lists, or visit to a breeder. Charity = website of a UK or foreign rehoming charity, or a visit to a rehoming centre. Commercial = pet selling websites, general selling websites, social media, local press, or local adverts. Personal = friends or family, local community or word-of-mouth

Table S8. – number of dogs owned per household and cumulative percentage.

Dogs per household	percent	Cumulative %
1	67.4	67.4
2	25.2	92.7
3	5.1	97.7
4 or more	2.3	100

Table S9. – Difference in percentage of sources of acquisition between 2018/2019 and the COVID-19 pandemic (2020/21).

Source	2018/2019	2020/2021	Difference
Pet websites	23.7	31.3	7.6
Family and friends	17.7	19.7	2
Foreign rehoming website	3.4	5.1	1.7
Breed group website	2.7	2.6	−0.1
Local adverts	0.2	0.1	−0.1
Local community	2.2	2.1	−0.1
Local press	0.5	0.2	−0.3
Breeder website	3.4	2.9	−0.5
Social media	5.5	4.7	−0.8
Kennel club website	6.3	5.2	−1.1
General website	8.9	7.5	−1.4
Breeder visit	5.6	4	−1.6
Rehoming websites	14.6	12.3	−2.3
Rehoming visit	5.2	2.2	−3
